# Effects of size and surface on the auxetic behaviour of monolayer graphene kirigami

**DOI:** 10.1038/srep35157

**Published:** 2016-10-12

**Authors:** Kun Cai, Jing Luo, Yiru Ling, Jing Wan, Qing-hua Qin

**Affiliations:** 1 College of Water Resources and Architectural Engineering, Northwest A&F University, Yangling, 712100, China; 2 Research School of Engineering, the Australian National University, Canberra, 2600, ACT, Australia

## Abstract

Graphene is an active element used in the design of nano-electro-mechanical systems (NEMS) owing to its excellent in-plane physical properties on mechanical, electric and thermal aspects. Considering a component requiring negative Poisson’s ratio in NEMS, a graphene kirigami (GK) containing periodic re-entrant honeycombs is a natural option. This study demonstrates that a GK with specific auxetic property can be obtained by adjusting the sizes of its honeycombs. Using molecular dynamics experiments, the size effects on the auxetic behaviour of GK are investigated. In some cases, the auxetic difference between the hydrogenated GK and continuum kirigami (CK) is negligible, in which the results from macro CK can be used to predict auxetic behaviour of nano kirigami. Surface effect of GK is demonstrated from two aspects. One is to identify the difference of mechanical responses between the pure carbon GK and the hydrogenated GK at same geometry and loading condition. Another is from the difference of mechanical responses between the GK model and the CK model under same loading condition and geometric configuration. Generally, surface energy makes the GK possess higher variation of auxetic behaviour. It also results in higher modulus for the GK as comparing with that of the CK.

When a sample of material is under tension or compression along longitudinal direction, it deforms not only along the loading direction but also along its lateral direction. The coupling between deformations along the sample’s loading and its lateral directions is governed by the Poisson’s ratio, which is defined as the negative ratio of the lateral normal strain to the longitudinal normal strain for a sample of material subjected to a longitudinal loading. The material is known as an auxetic material^1^, if the lateral normal strain is positive when the sample is subjected to a tension in longitudinal direction, or the lateral normal strain is negative when the sample is in compression in longitudinal direction. For an auxetic material, therefore, its Poisson’s ratio is negative. Auxetic materials exist widely in practical engineering. For example, 69% of the cubic metal crystals and some face-centred cubic rare gas solids behaved auxetic when they were stretched along the specific [110] off-axis direction^2^,^3^. Many natural materials have been found to be auxetic^4^,^5^,^6^. In man-made materials, auxetic materials are also popular. They can be found in honeycomb^7^, foams^8^,^9^,^10^ microporous polymers^11^, composites^12^,^13^,^14^, ceramic^15^,^16^ and origami^17^. And auxetic materials in molecular scale had been reported as early as two decades ago^18^.

Based on thermodynamic considerations of elastic strain energy, Poisson’s ratio varies within −1.0 to 0.5, for a natural material. However, the range can be extended wider for artificial materials, by providing specific microstructure in the matrix. In this way a conventional non-auxetic material can be converted into an auxetic material with specific value of Poisson’s ratio by such technology as tailoring^19^,^20^,^21^,^22^,^23^. Re-entrant cellular structures are typical tailored-materials with auxetic property, which have been well studied^6^. Their Poisson’s ratio as well as module rate varies when the sizes of some conformations of the structure, like the angle between struts and the dimensions of strut, change.

Auxetic materials are of interest in the field of material science because they exhibit the novel behaviour under deformation. It is possible to take advantage of the benefits of their negative Poisson’s ratio for a specific application by optimizing the microstructures of these materials, particularly in the rapidly developed nano-electro-mechanical systems (NEMS)^24^. Up to now, the design and synthesis of such auxetic materials at the molecular level is still a research focus^21^.

Single/few-layer graphene is a typical two dimensional (2D) isotropic material and has attracted much attention due to its excellent physical properties since it was obtained in laboratory^25^. For an ideal graphene, it is not an in-plane auxetics^5^. In recent years, graphene kirigami (GK) has been investigated due to its peculiar properties on deformation, electric conductivity and thermal conductivity^19^,^20^,^21^,^22^,^23^. As we tailor a kirigami of the 2D material into a re-entrant honeycomb (Fig. 1a) 6,26,27,28, auxetic behaviour will emerge. The question is, will the size effect still be significant on mechanical properties when the re-entrant cellular structure is miniaturized to nanoscale? Besides, will surface energy plays an important role in mechanical behaviour of the tailored GK like it in 2D material with caves? In present study, the deformation analysis of a continuum kirigami (CK) with its geometry matching that of GK is carried out using finite element method (FEM)^29^,^30^,^31^,^32^ aligning with molecular dynamics(MD) experiments of the GKs, aiming at revealing the influences of the sizes of microstructure and surface energy^33^,^34^,^35^ on the mechanical properties of such GK.

## Models and Methods

### Geometric model of kirigami

**Figure 1:**
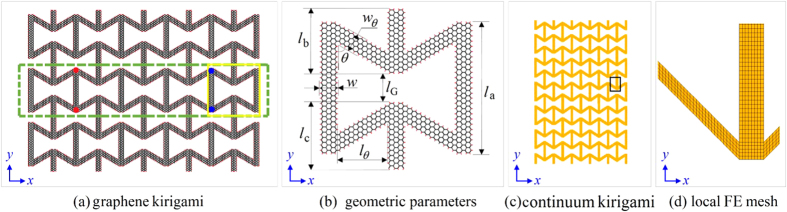
Schematic of a graphene kirigami with detailed geometric parameters. (**a**) The atomic system with periodic microstructure. (**b**) Geometry of local microstructure (in the solid yellow frame in (**a**)) with detailed geometric parameters of the kirigami. The microstructure has one more vertical rod than the unit cell. (**c**) The finite element model of the continuum kirigami. (**d**) The local finite element (FE) mesh in the solid black frame in (**c**).

The mechanical behaviour of the GK shown in Fig. 1a or continuum model shown in Fig. 1c 6,26,27,28 depends on surface energy distribution and five independent variables, i.e., *θ* (angle between oblique bar and vertical bar), *w*_*θ*_ (width of oblique bar), *w* (width of vertical bar), *l*_G_ (gap of adjacent vertical bars, it reflects the length of vertical bar) and *l*_*θ*_ (it reflects length of oblique bar). The deformation of the atomic system (in Fig. 1a) will be calculated using the result from MD simulation. The deformation of the CK (in Fig. 1c) will be solved via FEM. The engineering strain along y-direction is controlled within 5%. Hence, the equivalent modulus of kirigami can be calculated using the principle of minimum potential energy (*P*_e_). The engineering strain along x-direction, i.e., *ε*_*x*_, is calculated by the relative expansion of the blue points in Fig. 1a. The engineering strain along y-direction, i.e., *ε*_*y*_, is calculated by the time integration of the velocity of the upper side of specimen. The Poisson’s ratio in x-y plane is the ratio between −*ε*_*x*_ and *ε*_*y*_. To reflect the effect of surface energy on the equivalent modulus (in Eq. (4)), modulus ratio, i.e., the ratio between the equivalent modulus and the in-plane modulus of the two-dimensional matrix (i.e., the ideal graphene with modulus of ~995.8 GPa at 8 K), is defined as *R*_m_ in Eq. (5). The Poisson’s ratio and the modulus ratio of the atomic system and the continuum system will be compared to show the effect of surface energy. Geometric size effect is considered by varying the five independent geometric variables. The Poisson’s ratio and modulus ratio can be obtained using the following equations.


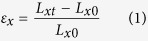



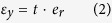



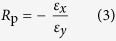



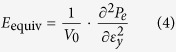



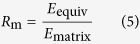


Where *L*_*x*0_ and *L*_*xt*_ are the initial and current horizontal distance of the red and blue points in Fig. 1a. *t* is the time of loading. *V*_0_ is the volume occupied by the atoms in statistic area for finding the potential energy of the atoms (*P*_e_). The thickness of graphene is set to be 0.335 nm in this study.

### Methods for numerical experiments

For the nanostructure of GK, the open sources for large scale MD simulator, LAMMPS^36^, is adopted. In simulation, the AIREBO^37^ potential function is used to describe the interaction among the hydrogen and carbon atoms. The time step for integration is 0.0005 ps. The procedure of MD experiment contains following steps:

Step 1) Build the kirigami model (within the green frame in Fig. 1a) with specified geometry. Once the hydrogenation model is adopted to the simulation, the red bond of edge in Fig. 1b will be bonded with hydrogen atoms;

Step 2) Set the two vertical sides (left and right sides) of the nanostructure as free boundaries, and the two horizontal sides as periodic boundaries (Let *l*_a_ = *l*_b_ + *l*_c_);

Step 3) Reshape the structure by minimizing the potential energy of the nanostructure. The conjugated-gradient algorithm is adopted for update the positions of the atoms;

Step 4) Relax the nanostructure in a Nosé-Hoover thermostat for 200 ps with temperature of 8 K;

Step 5) Provide a strain ratio of *e*_r_ = 0.005%/ps along y-direction on specimen. The total motion costs 1000 ps;

Step 6) Calculate the engineering strains along x- and y-directions according to the positions of the blue points in Fig. 1a. Calculate the Poisson’s ratio according to Eq. (3). Calculate the equivalent elastic modulus of the kirigami using Eq. (4). And find the ratio between the equivalent modulus and that of the ideal graphene.

To show the effect of surface energy on the mechanical properties of GK, hydrogenation schemes are added. If there is no carbon atom on the graphene bonded with hydrogen atom, the model is labelled as “+0H”; if each carbon atom on the edge (i.e., unsaturated carbon atom) is bonded with a hydrogen atom, the model is labelled as “+1H”. The potential energy and volume of the system with respect to the “+1H” model are the summation of those of carbon atoms only. All the results will be compared with those of a macrostructure with geometric similarity to the GK. The Poisson’s ratio and the modulus ratio of the structure of CK are analysed by finite element method. The analysis procedure of a deformed continuum using FEM includes the following steps:

Step a) Build the CK structure (shown in Fig. 1c) which has the same geometric configuration with that of the GK;

Step b) Mesh the CK structure with finite elements (plane stress elements);

Step c) Apply displacement constraints: the lower side is fixed along y-direction. The node located at the center of the lower side is fully fixed. Provide the nodes on the upper side of the structure with specified displacement, which reflects the same engineering strain along y-direction, i.e., no more than 5%;

Step d) Find the deformation of the CK structure by solving its equilibrium equation;

Step e) Calculate the Poisson’s ratio using the displacement of the labelled points in Fig. 1c and equation (3). Calculate the modulus ratio using equations (5).

### Schemes for size effect analysis

**Figure 2:**
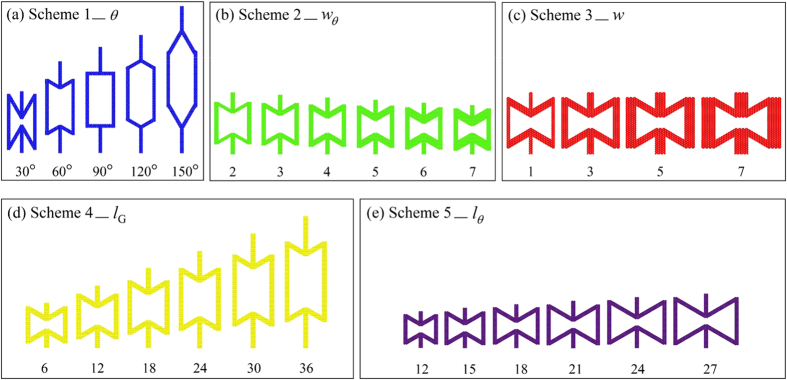
Configurations of the local microstructure of GK with different geometric parameters in five schemes. Here *N*(*w*) is denoted as the number of the basic C-C honeycomb cells along the direction of variable *w*, e.g., *N*(*w*) = 3 in Fig. 1b. (**a**) Scheme 1, *θ* changes; (**b**) *N*(*w*_*θ*_) changes; (**c**) *N*(*w*) changes; (**d**) *N*(*l*_G_) changes; (**e**) *N*(*l*_*θ*_) changes.

To show the size effect of the GK, the following schemes are considered:

Scheme 1) *θ* changes: Set *N*(*w*_*θ*_) = 3, *N*(*w*) = 3, *N*(*l*_G_) = 6, *N*(*l*_*θ*_) = 12, *N*(*l*_*a*_) = 55. *θ* changes from 30° to 60°, 90°, 120° and 150°. The microstructures are shown in Fig. 2a;

Scheme 2) *N*(*w*_*θ*_) changes: Set *θ* = 60°, *N*(*w*) = 3, *N*(*l*_*θ*_) = 12, *N*(*l*_*a*_) = 33. *N*(*w*_*θ*_) is chosen from {2, 3, 4, 5, 6, 7}, and the related value of *N*(*l*_*G*_) is set to be 16, 14, 12, 10, 8 and 6, respectively. The microstructures are shown in Fig. 2b;

Scheme 3) *w* changes: Set *θ* = 60°, *N*(*w*_*θ*_) = 3, *N*(*l*_G_) = 6, *N*(*l*_*θ*_) = 12, *N*(*l*_*a*_) = 25. *N*(*w*) is chosen from {1, 3, 5, 7}. The microstructures are shown in Fig. 2c;

Scheme 4) *l*_*G*_ changes: Set *θ* = 60°, *N*(*w*_*θ*_) = 3, *N*(*w*) = 3, *N*(*l*_*θ*_) = 12. *N*(*l*_G_) is chosen from {6, 12, 18, 24, 30, 36}, which leads *N*(*l*_*a*_) equal to 25, 31, 37, 43, 49 and 55, respectively. The microstructures are shown in Fig. 2d;

Scheme 5) *l*_*θ*_ changes: Set *θ* = 60°, *N*(*w*_*θ*_) = 3, *N*(*w*) = 3, *N*(*l*_G_) = 12. *N*(*l*_*θ*_) is chosen from {12, 15, 18, 21, 24, 27}, which makes *N*(*l*_*a*_) equal 25, 28, 31, 34, 37 and 40, correspondingly. The microstructures are shown in Fig. 2e.

## Theoretical analysis

**Figure 3:**
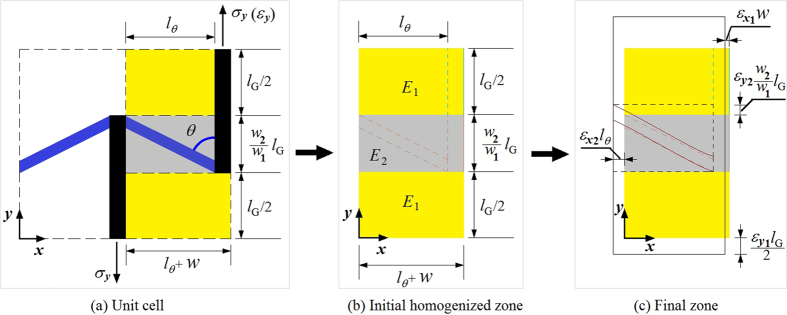
A part of the microstructure shown in Fig. 1b. The right half part of (**a**) is a quarter of the unit cell. The sizes are labelled. The grey area represents the domain whose mechanical property is determined by the oblique bar and the vertical bar. The mechanical property of the yellow area is determined by the vertical bar. (**b**) The homogenized model^38^ of the right half part of (**a**). *E*_1_ and *E*_2_ (<*E*_1_) are the in-plane equivalent moduli of the yellow and grey areas, respectively. (**c**) The final deformation.

To estimate the mechanical behaviour of GK, a theoretical analysis is introduced here. In Fig. 3, the right part with yellow and grey areas (including adjacent bars) is a quarter of the unit cell of kirigami. The equivalent mechanical properties, e.g., equivalent modulus and Poisson’s ratio are determined by the mechanical and geometric parameters of the oblique and vertical bars.

We suppose the total strain is along *y* direction, i.e., *ε*_y_, is specified and label the equivalent moduli of yellow and grey areas as *E*_1_ and *E*_2_, respectively. The two moduli can be obtained using MD simulation. The length weights of the two areas are *w*_1_ and *w*_2_ (=1 − *w*_1_), respectively. Hence, the equivalent modulus of the kirigami can be calculated using


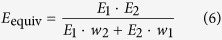


Let *ε*_y1_ and *ε*_y2_ represent the line strains of the yellow and grey areas along *y*-direction, respectively. They can be expressed as


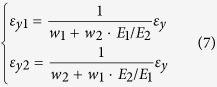


The line strain of the half part along *x*-direction, *x*-strain, can be calculated using:


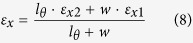


Where *ε*_*x*1_(<0) is the *x*-strain of the vertical bar adjacent to the yellow area. The value can be obtained using MD simulation. 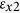 is the *x*-strain of the grey area. For the structure with small deformation, it can be obtained using the following formulation.


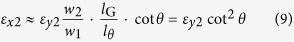


Hence, the equivalent Poisson’s ratio can be expressed as


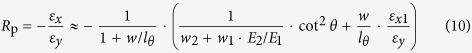


## Numerical results

### Results of Scheme 1_ θ varies

In this scheme, *θ* is chosen from the angles, i.e., 30, 60, 90, 120 and 150 degrees, respectively. The other parameters are given in Section “Schemes for size effect analysis”. After simulation, the Poisson’s ratio and the modulus ratio are calculated and shown in Table 1.

Table 1 lists the Poisson’s ratios and modulus ratios of the GK with different values of *θ*. For each Poisson’s ratio of GK in the three models, the value increases with the monotonous increase of *θ*. When *θ* is less than 90°, the Poisson’s ratio is negative, i.e., GK shows auxetic behaviour. At *θ* = 90°, the Poisson’s ratios are positive, rather than zero. The reason is that the oblique bar, which currently is horizontal, has a rotary angle which leads to *θ* > 90°. The table also demonstrates the value of *R*_p_ is sensitive to the surface energy. For example, for “+0H” model, *R*_p_ is 0.318, which is over twice of 0.149 of “+1H” model, and is far greater than 0.035 of CK model. Similar differences among the values of *R*_p_ of the three models can also be found at any other values of *θ*. It is concluded that the GK without hydrogenation (+0H model) has the highest absolute value of *R*_p_ among the three models. The hydrogenated GK (+1H model) has lower absolute value of *R*_p_ than the pure carbon kirigami does, but the value is higher than that of the CK (analysed using FEM) if *θ* is not 60°.

For the modulus ratios, *R*_m_, of kirigami in the three models, increase of them is seen with the increase of *θ*. The reason is that the deformation of the structure is caused by the rotation of the oblique bars when *θ* is lower, while, at higher value of *θ*, the deformation of GK is mainly caused by the axial linear strain of bars. Hence, increment of potential energy is higher at higher value of *θ*. According to Eq. (4), the modulus is higher when *θ* is greater. Comparing the three models’ modulus ratios at the same value of *θ*, the GK with “+1H” model has the highest modulus. It is about 10~20% higher than that of GK with “+0H” model. The reason can be found from the variation comparison of potential energy of the two models, i.e., in the “+1H” model, the carbon atoms on the edge have more steep increases as they are bonded with hydrogenation atoms. The modulus ratios of the GK with or without hydrogenation are far greater than that of the CK model under the same geometric parameter settings. It demonstrates that the surface influences the modulus of a 2D material significantly.

### Results of Scheme 2_ width of oblique bar varies

In this scheme, only *N*(*w*_*θ*_) changes from 2 to 7, indicating the width of oblique bars increases monotonously (Fig. 2b) while the other parameters are fixed. After simulation, the Poisson’s ratio and the modulus ratio are calculated and shown in Table 2.

When comparing *R*_p_ and *R*_m_ of the three models with the same geometric configurations, the surface effect can also be found. Similar conclusions as those in Scheme 1 can be found. Interestingly, the difference between *R*_p_ of GK with “+1H” model and that of the CK model is very small whatever the width of oblique bar is. It evidences that the auxetic behaviour of a 2D material shown in Fig. 2b can be estimated using experiment on a macro-continuum model.

Now, we examine the size effect on the mechanical properties of kirigami via adjusting the width of the oblique bars. Due to *θ* = 60° (less than 90°), the kirigamis of all the three models show auxetic behaviour when the width of oblique bar varies. It is also found that the absolute value of *R*_p_ decreases with the increase of the width of oblique bar. The reason can be explained from Eq. (10). In the model, the geometric parameters, e.g., *w*, *l*_*θ*_, *θ*, *l*_G_, and *E*_1_ and *ε*_y_ are constants. Both *w*_2_/*w*_1_ and *E*_2_ increase with the increase of the width of oblique bar. Negative scalar *ε*_*x*1_ decreases with the increase of the width of oblique bar. Hence, the absolute value of the *R*_p_ is lower when the width of oblique bar is wider. The modulus ratios of the three models are also different. The difference between the moduli of the GKs with and without hydrogenation is smaller than that between the GK models and CK model.

### Results of Scheme 3_ width of vertical bar varies

In this scheme, *N*(*w*) is chosen from 1, 3, 5, 7, i.e., the width of vertical bar increases monotonously. The rest parameters are fixed. After simulation, the Poisson’s ratio and the modulus ratio are calculated and shown in Table 3.

In the scheme, the absolute values of *R*_p_ do not vary monotonously, and the value peaks at *N*(*w*) = 3. The reason can be identified via the analysis with Eq. (10). For example, when *N*(*w*) = 1, the width of vertical bar is less than that of oblique bar, the vertical bar plays a major role in the deformation of GK. The rotary angle of the oblique bar is low because of small tension on the vertical bar (*σ*_*y*_). As *N*(*w*) = 3, the vertical bar has stronger tensile force which leads to higher rotary angle of oblique bar. Therefore, the *R*_p_ at *N*(*w*) = 3 is larger than that at *N*(*w*) = 1. When *N*(*w*) ≥ 5, the rotary angle of oblique bar is slightly higher than that at *N*(*w*) = 3. Simultaneously, *w*/*l*_*θ*_ is over 1.6 times of that at *N*(*w*) = 3. From the second item in the left part of Eq. (10), we can find that the wider width of the vertical bar (*N*(*w*) ≥ 5) leads to smaller Poisson’s ratio of GK. On the other hand, the difference between the Poisson’s ratios of GK with “+1H” model and CK model is obvious no matter if *θ* = 60° or not in the models, which implies that the surface effect cannot be neglected when using the continuum model to estimate the auxetic behaviour of GK with different widths of vertical bar.

For the modulus ratios of the three models, they decrease with the increase of width of vertical bar. The reason is that the deformation of GK mainly depends on the rotation of the oblique bar during loading along y-direction. Hence, the variation of potential energy of the vertical bar decreases with the increase of its width. Therefore, the variation of potential energy of system decreases, too, which results in decreasing of the modulus according to Eq. (4).

### Results of Scheme 4_ length of vertical bar varies

In this scheme, the length of vertical bar *N*(*l*_*G*_) is chosen from 6, 12, 18, 24, 30 and 36. The other parameters are fixed. After simulation, the Poisson’s ratio and the modulus ratio are calculated and shown in Table 4.

When the length of vertical bar increases or *N*(*l*_G_) changes from 6 to 36, the absolute values of Poisson’s ratio of the three models increase monotonously. The reason can be revealed by the analysis of either Eq. (10) or the deformation of Fig. 3b. For example, for the second item of the left part of Eq. (10), when *w*, *l*_*θ*_, *E*_1_ and *E*_2_(<*E*_1_), are constants, the increase of *w*_1_ leads to the increase of *R*_p_. It suggests that the 2D material can have higher auxetic effect by providing with longer vertical bars. In this scheme, the difference between the values of *R*_p_ of the hydrogenated GK and CK model is very small. One can estimate the axuetic behaviour of the GK using the experiments on the continuum model with the same geometric configurations according to this principle.

The modulus ratios of the three models increase with the increase of the length of vertical bar. But the maximal value is reached when *w*_1_ tends to be 1.0. The maximal value is *E*_1_/*E*_matrix_ (=(*w*/(*w*+*l*_*θ*_)) × (*E*_vert_bar_/*E*_matrix_) ≈20%) according to Eqs (5 and 6) and Fig. 3b. The hydrogenated GK still has the highest modulus among the three models. The CK model has the lowest modulus, which is no more than 20% of that of the hydrogenated GK.

### Results of Scheme 5_ length of oblique bar varies

In this scheme, the length of oblique bar varies, i.e., *N*(*l*_*θ*_) is chosen from 12, 15, 18, 24 and 27. The other parameters are fixed. After simulation, the Poisson’s ratio and the modulus ratio are calculated and shown in Table 5.

**Figure 4:**
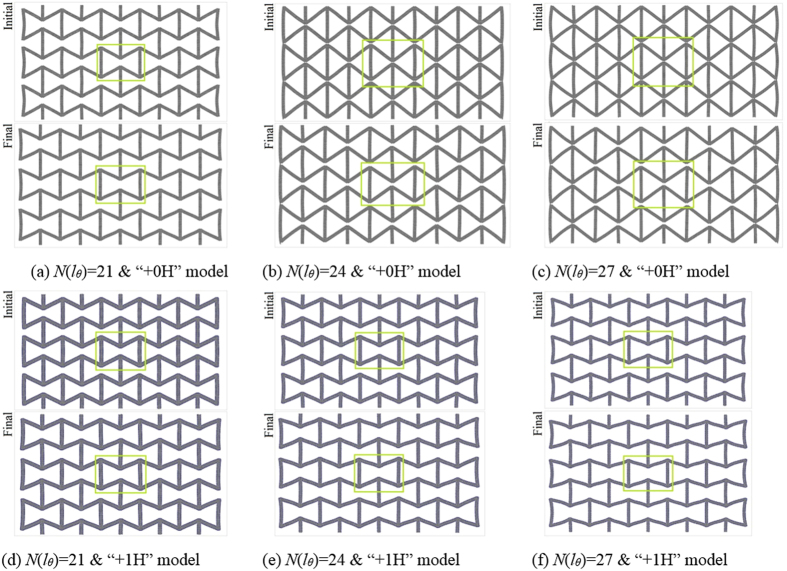
Comparison of configurations before and after deformation with respective to different lengths of oblique bar. In each inserted figure, the mid microstructure is labelled with a solid lime frame. In each case, the configuration of the structure is formed with three layers of microstructures. The upper figure in each case is the initial configuration after relaxation, the lower figure in each case is the final stable configuration after loading and relaxation.

**Figure 5:**
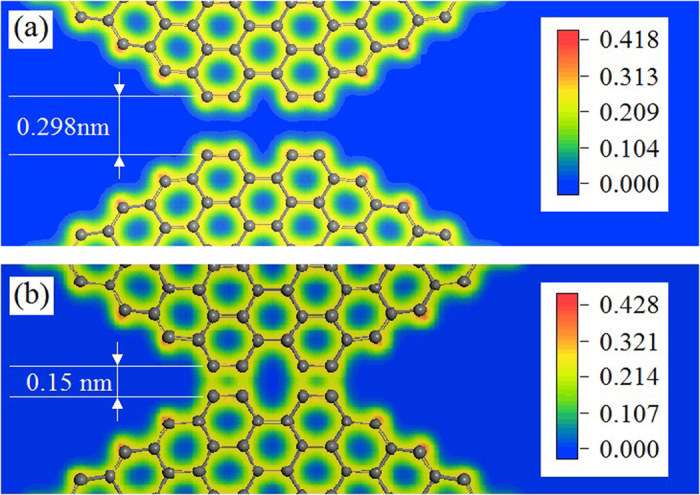
Different contour plot of electron density nearby the neighbour ends of vertical bars in the initial configuration of the pure carbon GK with (**a**) *N*(*l*_*θ*_) = 24 (see the upper layer of Fig. 4b) and (**b**) *N*(*l*_*θ*_) > 36 obtained by the first principles calculation^39^.

The results of *R*_p_ of CK with different geometric parameters show that the absolute value of *R*_p_ decreases slightly with the increase of the length of oblique bar. Although the similar surface effects still exist, demonstrated by the absolute value of *R*_p_ of hydrogenated GK is lower than that of the pure carbon GK, but greater than that of CK model, the value of *R*_p_ does not change monotonously with respect to the variation of the length of oblique bar. For example, the absolute value of pure carbon GK peaks at *N*(*l*_*θ*_) = 24. While, the maximal absolute value of hydrogenated GK appears at *N*(*l*_*θ*_) = 21. The reason can be revealed from the configurations of the models shown in Fig. 4. For instance, at *N*(*l*_*θ*_) = 21, the two vertical bars on the same column are not attracted to be bonded together. When *N*(*l*_*θ*_) = 24 or 27, the two vertical bars get close to each other, with the distance of only 0.298 nm for *N*(*l*_*θ*_) = 24 (Fig. 5a) or 0.265 nm for *N*(*l*_*θ*_) = 27. The two adjacent ends of vertical bars are not bonded. However, the van der Waals interaction between the two adjacent ends becomes very strong, which leads to the decrease of the value of *θ*. Hence, the Poisson’s ratio at *N*(*l*_*θ*_) = 24 is greater than that at *N*(*l*_*θ*_) = 21. When *l*_G_ keeps unchanged, larger *l*_*θ*_ leads to smaller variation of *θ* after relaxation. That is why the Poisson’s ratio at *N*(*l*_*θ*_) = 24 is higher than that at *N*(*l*_*θ*_) = 27. The electron density distribution nearby the adjacent ends of vertical bars shown in Fig. 5a reveals that no new carbon-carbon covalent bond is generated. Hence, after deformation, the two ends are separated. However, the variation of potential energy is greater than that of the model with *N*(*l*_*θ*_) = 21. It results in higher modulus according to Eq. (4). This is verified by the values of the *R*_m_ listed in Table 4. On the other hand, if the value of *N*(*l*_*θ*_) is far greater than 24, the oblique bars are softer and the two ends of the vertical bars can get closer, which provide a chance to generate new covalent bonds among the unsaturated carbon atoms on the ends (Fig. 5b). In such condition, the topology of the kirigami changes and the new GK may alter mechanical properties, such as becoming stiffer or even non-auxetic.

When the GK is hydrogenated (“+1H” model), the repulsion exists between the adjacent vertical bar and oblique bar at the joint whilst attraction exists between the two adjacent vertical bars. A model with longer oblique bar will have higher deformation of the oblique bar due to repulsion between the oblique and vertical bars and stronger attraction at adjacent ends of vertical bars. Hence, the peak value of Poisson’s ratio appears at *N*(*l*_*θ*_) = 21. On the other hand, the two adjacent ends of vertical bars are attracted due to van der Waals interaction, rather than covalent bonds, the potential energy of the system changes slightly. The modulus ratio decreases with the increase of *N*(*l*_*θ*_). The modulus ratio of CK model has similar variation. But the modulus ratio of hydrogenated GK is still much higher than that of CK.

## Conclusions

After analysis the Poisson’s ratio and modulus ratio of GK with re-entrant honeycomb microstructure, the dependence of the two mechanical properties on the sizes of the microstructure is revealed. And the mechanical response of the CK with geometric similarity to the GK is also calculated for finding the surface effect of GK through comparisons of the responses. The results show that the specified Poisson’s ratio and modulus of GK can be obtained by adjusting the sizes of microstructure. Some conclusions can be drawn as follows

1. Of all the schemes, pure carbon GK, being with the highest surface energy, has the highest absolute value of Poisson’s ratio among the three models. The absolute value of Poisson’s ratio of the hydrogenated GK is higher than that of the CK due to the higher surface energy of the GK than that of the CK. Considering the effect of θ in scheme 1, when *θ* is less than 90°, GK shows auxetic.
2. In general, the modulus ratio of hydrogenated GK is about 10~20% higher than that of the pure carbon GK. The modulus ratio of CK is far less than that of the GK when they have the same geometric configurations. It demonstrates the surface influence on the modulus of 2D nano materials.
3. In Scheme 2, the difference between the Poisson’s ratios of hydrogenated GK and CK models is very small, meaning that the Poisson’s ratios depend on the width of oblique bar, slightly. It indicates that the auxetic behaviour of a 2D nanomaterial can be estimated using the experiment on a macro-continuum model with respect to the width of oblique bar. For the modulus ratios of GK and CK models, when the width of oblique bar increases, the absolute value of *R*_p_ decreases, but the modulus ratio increases, monotonically.
4. When increasing the width of vertical bar like in scheme 3, the peak value of Poisson’s ration appears at *N*(*w*) = 3. The difference between the Poisson’s ratios of GK models and CK model is obvious. As for the modulus ratios of the three models, they decrease with the increase of width of vertical bar.
5. When the length of vertical bar *N*(*l*_G_) increases from 6 to 36, the absolute values of Poisson’s ratio of the three models increases monotonously. It suggests that the 2D material can have higher auxetic effect with longer vertical bars as in Scheme 4. In this condition, the difference between the values of Poisson’s ratio of the hydrogenated GK and CK model is very small. The axuetic behaviour of GK can be estimated using the experiments on the CK model with the same geometric configurations. The modulus ratios of the three models increase with the increase of the length of vertical bar.
6. In Scheme 5, the absolute value of Poisson’s ratio varies slightly with the increase of the length of oblique bar (or *N*(*l*_*θ*_)). If the value of *l*_G_ is small (i.e., vertical bars are short), the two adjacent vertical bars in pure carbon GK model may bonded together. It results in the sharp increase of both Poisson’s ratio and modulus ratio. But it does not happen in either the hydrogenated GK model or the CK model.

## Additional Information

**How to cite this article**: Cai, K. *et al.* Effects of size and surface on the auxetic behaviour of monolayer graphene kirigami. *Sci. Rep.* **6**, 35157; doi: 10.1038/srep35157 (2016).

## Figures and Tables

**Table 1:** Comparisons of Poisson’s ratio and the modulus ratio for the three models in Scheme 1.

	*θ* = 30°	*θ* = 60°	*θ* = 90°	*θ* = 120°	*θ* = 150°
*R*_p_ (GK + 0H)	−2.074	−0.980	0.318	1.868	6.302
*R*_p_ (GK + 1H)	−1.442	−0.669	0.149	1.667	5.077
*R*_p_ (CK)	−1.139	−0.722	0.035	1.034	2.907
*R*_m_ (GK + 0H)	5.16%	14.40%	17.91%	23.14%	31.27%
*R*_m_ (GK + 1H)	7.08%	16.86%	22.31%	25.63%	37.91%
*R*_m_ (CK)	1.88%	2.05%	2.19%	2.59%	4.47%

• The in-plane modulus of the ideal graphene at 8 K is ~995.8 GPa.

**Table 2:** Comparisons of Poisson’s ratio and the modulus ratio for the three models in Scheme 2.

	*N*(*w*_*θ*_) = 2	*N*(*w*_*θ*_) = 3	*N*(*w*_*θ*_) = 4	*N*(*w*_*θ*_) = 5	*N*(*w*_*θ*_) = 6	*N*(*w*_*θ*_) = 7
*R*_p_ (GK + 0H)	−0.835	−0.593	−0.470	−0.333	−0.235	−0.171
*R*_p_ (GK + 1H)	−0.558	−0.457	−0.336	−0.239	−0.171	−0.119
*R*_p_ (CK)	−0.517	−0.413	−0.311	−0.226	−0.163	−0.118
*R*_m_ (GK + 0H)	3.96%	6.54%	8.21%	9.43%	9.91%	9.92%
*R*_m_ (GK + 1H)	6.41%	7.96%	9.08%	10.74%	11.10%	11.34%
*R*_m_ (CK)	0.47%	1.16%	2.04%	2.94%	3.75%	4.46%

**Table 3:** Comparisons of Poisson’s ratio and the modulus ratio for the three models in Scheme 3.

	*N*(*w*) = 1	*N*(*w*) = 3	*N*(*w*) = 5	*N*(*w*) = 7
*R*_p_ (GK + 0H)	−0.406	−0.455	−0.416	−0.372
*R*_p_ (GK + 1H)	−0.311	−0.345	−0.337	−0.305
*R*_p_ (CK)	−0.256	−0.280	−0.254	−0.225
*R*_m_ (GK + 0H)	4.67%	3.58%	2.82%	2.36%
*R*_m_ (GK + 1H)	5.56%	4.35%	3.46%	3.35%
*R*_m_ (CK)	0.83%	0.79%	0.68%	0.60%

**Table 4:** Comparisons of Poisson’s ratio and the modulus ratio for the three models in Scheme 4.

	*N*(*l*_G_) = 6	*N*(*l*_G_) = 12	*N*(*l*_G_) = 28	*N*(*l*_G_) = 24	*N*(*l*_G_) = 30	*N*(*l*_G_) = 36
*R*_p_ (GK + 0H)	−0.455	−0.559	−0.662	−0.763	−0.845	−0.980
*R*_p_ (GK + 1H)	−0.345	−0.430	−0.505	−0.560	−0.623	−0.669
*R*_p_ (CK)	−0.280	−0.381	−0.475	−0.563	−0.645	−0.722
*R*_m_ (GK + 0H)	3.58%	5.71%	7.94%	9.97%	12.29%	14.40%
*R*_m_ (GK + 1H)	4.35%	6.93%	9.58%	11.93%	14.36%	16.86%
*R*_m_ (CK)	0.79%	1.07%	1.34%	1.59%	1.82%	2.05%

**Table 5:** Comparisons of Poisson’s ratio and the modulus ratio for the three models in Scheme 5.

	*N*(*l*_*θ*_) = 12	*N*(*l*_*θ*_) = 15	*N*(*l*_*θ*_) = 18	*N*(*l*_*θ*_) = 21	*N*(*l*_*θ*_) = 24	*N*(*l*_*θ*_) = 27
*R*_p_ (GK + 0H)	−0.455	−0.484	−0.437	−0.431	−0.551	−0.544
*R*_p_ (GK + 1H)	−0.345	−0.362	−0.377	−0.383	−0.350	−0.305
*R*_p_ (CK)	−0.280	−0.278	−0.271	−0.264	−0.256	−0.249
*R*_m_ (GK + 0H)	3.58%	2.16%	1.41%	1.17%	5.03%	9.99%
*R*_m_ (GK + 1H)	4.35%	2.82%	1.92%	1.61%	1.26%	0.90%
*R*_m_ (CK)	0.79%	0.42%	0.25%	0.16%	0.11%	0.08%
